# Functional Mechanisms of Recovery after Chronic Stroke: Modeling with the Virtual Brain^1^,^2^,^3^

**DOI:** 10.1523/ENEURO.0158-15.2016

**Published:** 2016-04-04

**Authors:** Maria Inez Falcon, Jeffrey D. Riley, Viktor Jirsa, Anthony R. McIntosh, E. Elinor Chen, Ana Solodkin

**Affiliations:** 1 Department of Anatomy and Neurobiology, UC Irvine School of Medicine, Irvine, California 92697; 2 Institut de Neurosciences des Systèmes, Aix-Marseille Université, Faculté de Médecine, Marseille F-13000, France; 3 Inserm UMR1106, Marseille F-13000, France; 4 Rotman Research Institute, Baycrest Health Sciences, M6A 2E1 University of Toronto, Toronto, Canada; 5 Department of Neurology, UC Irvine School of Medicine, Irvine, California 92697

**Keywords:** brain dynamics, brain networks, computational biophysical modeling, connectome, imaging, stroke

## Abstract

We have seen important strides in our understanding of mechanisms underlying stroke recovery, yet effective translational links between basic and applied sciences, as well as from big data to individualized therapies, are needed to truly develop a cure for stroke. We present such an approach using The Virtual Brain (TVB), a neuroinformatics platform that uses empirical neuroimaging data to create dynamic models of an individual’s human brain; specifically, we simulate fMRI signals by modeling parameters associated with brain dynamics after stroke.

In 20 individuals with stroke and 11 controls, we obtained rest fMRI, T1w, and diffusion tensor imaging (DTI) data. Motor performance was assessed pre-therapy, post-therapy, and 6–12 months post-therapy. Based on individual structural connectomes derived from DTI, the following steps were performed in the TVB platform: (1) optimization of local and global parameters (conduction velocity, global coupling); (2) simulation of BOLD signal using optimized parameter values; (3) validation of simulated time series by comparing frequency, amplitude, and phase of the simulated signal with empirical time series; and (4) multivariate linear regression of model parameters with clinical phenotype. Compared with controls, individuals with stroke demonstrated a consistent reduction in conduction velocity, increased local dynamics, and reduced local inhibitory coupling. A negative relationship between local excitation and motor recovery, and a positive correlation between local dynamics and motor recovery were seen.

TVB reveals a disrupted post-stroke system favoring excitation-over-inhibition and local-over-global dynamics, consistent with existing mammal literature on stroke mechanisms. Our results point to the potential of TVB to determine individualized biomarkers of stroke recovery.

## Significance Statement

The development of schemes to acquire neuroimaging big data is fostering a greater understanding of brain function. Yet we are lacking quantitative tools to translate these insights to the individual level, particularly associated with neurological disease. We address this challenge using the neuroinformatics platform, The Virtual Brain, to model individualized brain activity. This approach enables the linkage of macroscopic brain dynamics with mesoscopic biophysical parameters, wherein we demonstrate the capacity of large-scale brain models to track and predict long-term recovery after stroke. Our results establish the basis for a deliberate integration of computational biology and neuroscience into clinical approaches for elucidating cellular mechanisms of disease, opening new venues for the development of individualized therapeutic interventions.

## Introduction

Previous research has provided key insights into the disease process in stroke. Studies in mammals have uncovered basic mechanisms of ischemic injury, inflammatory responses, and cellular recovery (Carmichael, 2012; Nudo, 2013). In humans, researchers have suggested predictive imaging biomarkers for disease progression and recovery, mapped associated changes in brain networks, and developed new rehabilitative therapies (Reiss et al., 2012). Despite this, stroke remains a major source of disability in the United States, with ∼6.5 million people living with stroke, with some level of hemiparesis present in ∼50% (Go et al., 2014). This is neither the fault of mammal nor human studies, as both are constrained by their respective study populations. Studies in mammals are well controlled yet homogeneous, limiting their translational abilities. Human studies reflect the population at hand, yet often rely on indirect measures, obscuring the full picture. Although both share a common goal of curing stroke via the repair and reorganization of the injured brain, what is missing is a translational bridge to effectively span the divide between basic mechanisms and dynamic human brain systems.

At the same time, the neuroscience community is immersed in collecting large datasets to provide greater understanding of brain function and dysfunction. Such initiatives span normal function (Human Connectome Project), development (NIH Pediatric Database), and brain disorders, such as Alzheimer’s disease (ADNI) and mental illness (Research Domain Criteria Project). Although these initiatives provide the necessary empirical foundation, quantitative tools are missing to integrate these multiple datasets to “reconstruct” the brain, and provide the link between these data and those from a single person.

Over the last 6 years, a neuroinformatics platform has been developed: The Virtual Brain (TVB) (Sanz Leon et al., 2013). The defining feature of TVB is that it generates personalized functional neuroimaging data based on individual structural connectome data to create personalized virtual brains. These models are specific to each individual person, and contain the connectivity between parts of the brain and the dynamics of local neural populations. TVB uses structural MRI data to create the custom brain surface, diffusion-weighted MRI data to infer the anatomical connections between brain areas, and then functional MRI data as the target to modify the parameters of the model to reproduce the observed functional data. The neuroinformatics architecture of TVB houses a library of models, which catalogues the biophysical parameters that produce different empirical brain states (Ritter et al., 2013). Global biophysical parameters represent biological mechanisms governing dynamics between brain regions, whereas the local biophysical parameters describe the properties of small populations of neurons integrating dynamics at the local mesoscopic level. That is, modeling in TVB comprises multiple scales of brain dynamics that are invisible to brain imaging devices, and therefore TVB acts as a “computational microscope”, allowing the inference of internal states and processes of the system.

TVB thus offers a novel platform to formulate biologically interpretable hypotheses on the effects of stroke and its recovery based on biophysical mechanisms governing brain dynamics. Beyond the direct clinical implications of network dysfunction in stroke, these insights can contribute a first step to the understanding of fundamental mechanisms of the brain’s structure–function relationship. TVB has been established and applied to normative datasets (Deco et al., 2012) and for learning and plasticity (Roy et al., 2014), yet a proof of concept needs to be established based on pathological states.

The objective of the present study using the TVB platform was to determine changes in local and global biophysical parameters to better understand individualized brain dynamics after stroke. In this approach, the model parameters act as a means to assess brain health, analogous to blood samples assessing physical health, and hence, parameter changes could ideally be used as potential biomarkers of stroke and/or stroke recovery. So far, such biomarkers have mostly focused on stable architectures, from behavior to fine anatomical and functional levels (Burke and Cramer, 2013). In contrast, our aim is to create a synergistic amalgamation of mathematical models with neuroimaging, where the biomarker derives from the dynamical model itself.

## Methods

### Subjects

Twenty volunteers with chronic stroke (ages 23–74, 8 females) in the middle cerebral artery (MCA) territory and 11 age-matched controls were included in the study. This study and all procedures for recruitment and consent were approved by the Institutional Review Board of the University of Chicago and the University of California Irvine Medical School. Demographic details and stroke characteristics of our cohort can be found in Table 1.

**Table 1.** Demographics and stroke characteristics of the stroke cohort

Subject	Age	Sex	Handedness	Affected hemisphere	Affected hand	Stroke location	Stroke volume,mm^3^
1	41	F	Right	Right	ND	Cort	22495.0
2	54	F	Right	Left	D	Cort/subcort	49078.0
3	57	M	Right	Left	D	Cort/subcort	17411.0
4	57	M	Right	Left	D	Cort/subcort	38703.0
5	54	F	Right	Left	D	Subcort	27677.0
6	50	M	Right	Right	ND	Subcort	3570.0
7	23	M	Right	Left	D	Subcort	560.0
8	55	F	Right	Right	ND	Cort	6781.0
9	68	M	Right	Left	D	Subcort	1988.3
10	56	F	Right	Left	D	Subcort	6239.7
11	46	M	Right	Left	D	Subcort	325.0
12	56	F	Left	Right	D	Cort/subcort	60669.0
13	37	M	Right	Left	D	Cort/subcort	83406.2
14	62	M	Right	Left	D	Subcort	22154.8
15	57	M	Right	Right	ND	Cort/subcort	25392.0
16	66	M	Right	Left	ND	Cort/subcort	19927.0
17	61	M	Right	Left	D	Subcort	978.0
18	74	M	Right	Left	D	Cort/subcort	63642.0
19	67	F	Right	Right	ND	Subcort	588.0
20	74	F	Right	Left	D	Cort/subcort	44892.0

D, Dominant hemisphere; ND, non-dominant hemisphere; Cort, cortical, Subcort, subcortical.

Motor performance was assessed with the following: the Functional Ability Scale of the Wolf Motor Function Test (WMFT), Nine-hole peg test, the Fugl–Meyer upper arm test, and the Motor Activity Log (MAL-14). These assessments were collected at baseline (pre-therapy), after 1 month of intensive hand therapy (post-therapy) and 6–12 months after therapy (maintenance).

### Brain imaging

Imaging data were acquired on a 3 Tesla Philips Achieva scanner using the following sequences:

(1) High-resolution anatomical images were acquired with a 3D magnetization-prepared rapid acquisition gradient echo (MP-RAGE) sequence: FOV= 250 × 250, resolution=1 × 1×1 mm, SENSE reduction factor =1.5, TR/TE=7.4/3.4 ms, flip angle=8, sagittal orientation, number of slices=301 covering the whole brain.

(2) Diffusion tensor imaging (DTI) was acquired with the following sequence: FOV=224 × 224, TR/TE=13030/55, 72 slices, slice thickness= 2 mm, resolution=0.875 × 0.875 × 2, 2 mm post-processing iso-voxel with *b*=1000 s/mm^2^ (and *b*=0), 32 diffusion directions.

(3) Functional imaging acquisition at rest covering the whole brain (37 slices) was acquired using single-shot echo-planar MR (EPI) with slice thickness = 4.0 mm, FOV= 230 × 230, voxel size = 2.8× 2.8 mm, TR/TE= 2000/20 ms, duration= 5 min.

### Virtual brain transplantation

Because of mechanical deformation consequent to large cortical strokes, the anatomical parcellation on T1w images using semiautomated methods is very difficult to achieve. Hence, a “virtual brain transplant” process was performed in accordance with a previous approach (Solodkin et al., 2010). This method replaces the cortical lesion with the homologous image from the contralesional hemisphere from the same subject. With this, brain parcellation is possible using semiautomatized software. The process consisted of the following steps:

(1) Lesion segmentation by hand. (2) Using the AFNI 3dcalc function (Cox, 1996), the homologous region in the nonlesioned hemisphere was dissected and transplanted into the stroke region, effectively filling in the missing portions of the brain. (3) Manual corrections were then done in the interface between the native and transplanted T1-w images by visually examining each voxel and making voxel intensities uniform using AFNI’s 3dLocalStat and 3dcalc commands. (4) The brain was then parcellated into 96 cortical and subcortical regions. The original parcellation based on a macaque template (Van Essen, 2004) was transformed to the human MNI template via PALS (Van Essen, 2005). To increase accuracy, the deformation process was carried out using landmarks (based on CARET) and functional activation patterns considered homologous between the two species (Van Essen and Dierker, 2007).

### Diffusion tensor imaging

Preprocessing of DTI data consisted of the following: (1) motion correction using the FSL eddy current correction (Leemans and Jones, 2009), (2) generation of a binary brain mask from the b0 image and application of the mask to all diffusion images using the Brain Extraction Tool from FSL (Smith, 2002), (3) fitting of a diffusion tensor at each voxel using FSL’s dtifit function, (4) nonlinear coregistration of T1 data to the MNI brain and coregistration of T1 images to their respective DTI images producing an MNI to DTI transformation using ANTS (Avants et al., 2011), (5) white and gray matter segmentation performed on the MNI-to-T1 atlas using FAST (Zhang et al., 2001), and (6) parcellation of the gray matter into 96 regions as described above and registration of these regions to the DTI using the T1-to-DTI transformation with a nearest neighbor interpolation.

### Tractography and structural connectivity matrix generation

Probabilistic tractography was performed to trace the fiber bundles associated with pairs of cortical regions in the MNI space, which were defined as edges in the network (Ritter et al., 2013; Zalesky and Fornito, 2009).Two connectivity measures were extracted: (1) capacities, depicting the maximum rate of transmission of information through edges, were calculated using the number of streamlines at the minimum cross-sectional area of an edge (Zalesky and Fornito, 2009); and (2) distances, defined by the lengths of each edge, were calculated by averaging the lengths of all streamlines in an edge. These measures were used to generate two 96 × 96 structural connectivity matrices. Quality assurance to reduce false-positives was performed on each structural connectivity matrix by a trained neuroanatomist (A.S.).

### Resting state fMRI preprocessing

Preprocessing was done in AFNI (Cox, 1996) and included the following steps: motion correction of functional and anatomical datasets (Cox and Jesmanowicz, 1999), 3D spatial registration to a reference acquisition from the rsfMRI run, registration of functional images to the T1-w volume, de-spiking and mean normalization of the time series, motion correction (>1 mm; Johnstone et al., 2006) and regression of CSF and white matter signals to remove slow-wave components (eg, physiological noise; Lund et al., 2006).

### Resting state fMRI postprocessing

Average time series were extracted for each of 96 MNI regions. For each subject, a 96 × 96 functional connectivity matrix was generated by calculating the pairwise correlation of the time series for each region (Ritter et al., 2013) using the “corr” function in MATLAB.

### Modeling in TVB

**Figure 1.**
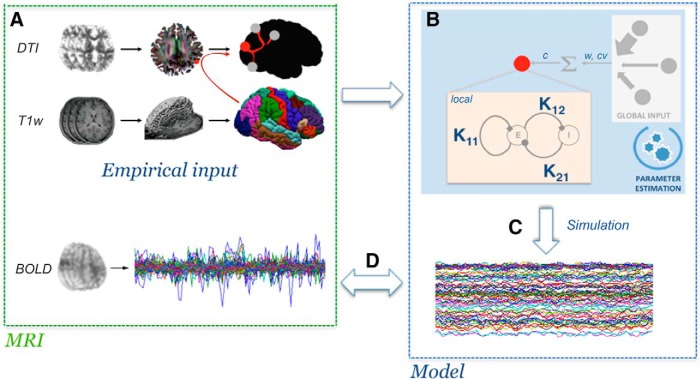
Simulation workflow in TVB. Graphic representation depicting the sequential steps of TVB modeling. ***A***, Empirical inputs (structural connectome) are generated from DTI tractography based on T1-w brain parcellation. ***B***, Subsequent parameter exploration at the global and local levels (w, Weights; cv, conduction velocity; c, global coupling). ***C***, Once parameter values are obtained, the BOLD signal is simulated. ***D*,** The efficacy of the simulation is calculated by correlating it to the empirical signals.

**Figure 2.**
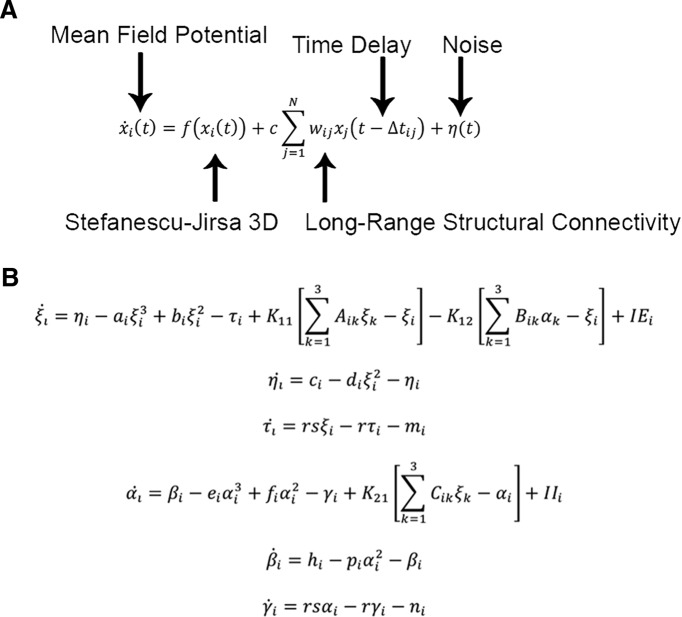
Equations of the Stefanescu-Jirsa 3D model. ***A***, Evolution equation implemented in TVB to simulate brain activity. The mean field potential *x_i_*(*t*) of a region *i* at time *t* is dependent on the local dynamics *f*(*x_i_*(*t*)) provided by the Stefanescu-Jirsa-3D model, the long-range structural connectivity w, which links regions *i* and *j* and is provided by the input of individual structural connectivity matrices (weights), and noise η(t). Time delays (Δt) are distance dependent and are provided by the structural connectivity matrices (lengths). All mathematical details of the model and its numerical implementation are provided by Sanz-Leon et al. (2015). ***B***, Equations comprising Stefanescu-Jirsa 3D. The first three equations (ξ, η, τ) represent the excitatory subpopulation of neurons within a local region, whereas the last three equations (α, β, γ) represent the inhibitory subpopulation of neurons in that region. IE and II denote the input current to the excitatory and inhibitory populations of each node, respectively. The first of each of the two sets of equations accounts for neuron potentials. The second and third equations account for the transport of ions across the membrane through ion channels. Note that the dynamics of these populations are dependent on the interactions between inhibitory and excitatory influences (*K*_12_, *K*_21_, *K*_11_).

The Virtual Brain (TVB v1.08; Fig. 1) was used for all simulations (Sanz Leon et al., 2013) where the principal empirical input to the platform is the structural connectivity matrix derived from each individual subject’s tractography. Based on this input, TVB simulates field potentials by integrating global dynamics with a local (mesoscopic) model that determines the dynamics within brain regions. Following, BOLD signals are derived from the generated field potentials. In this work, we used the Stefanescu-Jirsa 3D (SJ3D; Fig. 2) local model, as the resulting mean field model does not rely heavily on synaptic delays (Stefanescu and Jirsa, 2008; Jirsa and Stefanescu, 2011; Sanz-Leon et al., 2015), making it compatible with the poor time resolution associated with BOLD signals. Specifically, the SJ3D model is derived from populations of bursting neurons and includes six states describing excitatory and inhibitory dynamics via the inclusion of a variety of biophysical parameters defining the local mean fields (for a list of the parameter values used in the present study see Table 2; Hindmarsh and Rose, 1984; Stefanescu and Jirsa, 2008).

**Table 2.** State variables and parameters of the Stefanescu-Jirsa 3D model and corresponding range of values used in the present study

Parameter	Value	Description
*a*, *b*, *c*, *d*	1, 3, 1, 5	Constants affecting faster ion channels
*r*	0.006	Constant affecting slower ion channels
*s*	4	Bursting strength of model
μ and σ	2.2, 0.3	Mean and dispersion of input current in each node
X_0_	−1.6	Leftmost equilibrium point of X
IE, II	Derived from μ and σ	Models excitability of each node and mode (IE for excitatory input, II for inhibitory input)
Global Coupling	0.01–1.0	Coupling scaling factor for connections between nodes
Conduction velocity	10–100	Scales delay for defined internode distances
β, γ	4, 5	Corresponding values for IPs
*K*_12_,*K*_21_,*K*_11_	0.01–1.0	Models coupling between excitatory and inhibitory populations within nodes

Values used for the simulation included global coupling, conduction velocity, and *K*_12_, *K*_21_, and *K*_11_ optimized via parameter space explorations. Default values were used for all other variables.

**Figure 3.**
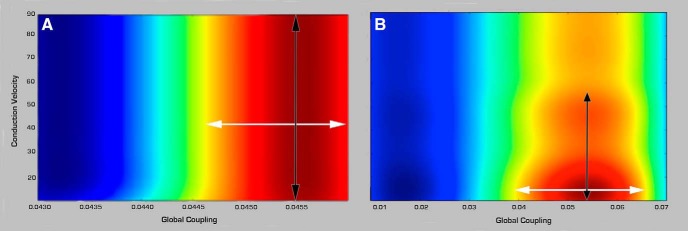
Examples of global parameter space explorations in healthy controls and stroke. Two examples of heat graphs of global variance (mean variance of the time series across all regions) used to narrow down the range of parameter values more suitable for modeling in (***A***) a healthy control and (***B***) a stroke case. Global coupling is shown on the *x*-axis and conduction velocity (m/s) on the *y*-axis. Colors indicate degree of global variance with hotter colors indicating higher values. White arrows show the range of values considered for global coupling limited by bifurcation points (yellow). Black arrows point to the range in conduction velocity considered in each case. Note the higher range of values associated with global coupling and lower for conduction velocity in the stroke case.

The following sequential steps were performed for each individual subject:

(1) Importing of a subject-specific connectivity matrix into the TVB platform.

(2) Selection of the SJ3D local model.

(3) Parameter space estimation (exploration and fitting). We sequentially performed systematic parameter space explorations and fitting to determine the optimal values for global and local parameters in all subjects. (1) Parameter space exploration: we used heat maps of global variance (mean variance of time series across all brain regions) to constrain the range of values for each model parameter (Fig. 3). The range of values considered is assessed based on those values with high global variance flanked by bifurcation points (Breakspear and Jirsa, 2007). An additional advantage of this approach is that it is not only pragmatic but it can also provide information on the degree of variability and sensitivity that parameter values have onto the simulated signals. (2) Parameter fitting: the final optimal value was subsequently obtained by assessing the specific value for the parameters that resulted in the best fit between the empirical and simulated signals based on three metrics described below (Step 6). The global parameters explored included conduction velocity and global coupling and the local parameters included *K*_12_ (excitatory on inhibitory coupling), *K*_21_ (inhibitory on excitatory coupling), and *K*_11_ (excitatory on excitatory coupling). The local parameters were chosen as they have the strongest impact on the dynamics of the SJ3D model (Stefanescu and Jirsa, 2008).

(4) Stochastic network simulation: based on the values obtained in the parameter space exploration, we generated field potentials with the same duration (4 min) and sampling rate (TR=2 s) as the empirical rsfMRI acquisition. The length of the simulated data was kept equal to the length of the empirical data to minimize the influence of variability over the course of the time series, as it is becoming increasingly patent that values of functional connectivity are not stable over time (Hutchison et al., 2013). White noise with Gaussian amplitude (mean = 0, SD = 1) was added to each node. Numerical integration of the system was performed using stochastic Heun’s method (Mannella 2002), with an integration step size of 0.0122 ms.

(5) The BOLD signals were derived from the field potentials using a hemodynamic response function implemented with a gamma kernel (Boynton et al., 1996; Sanz-Leon et al., 2015).

(6) Assessing reliability of the simulated time series: comparison between the empirical and simulated BOLD time series was done in terms of amplitude, frequency, and phase. Amplitude: we calculated the range of amplitude by identifying the highest and lowest peaks present in the time series across all regions. Frequency: fast Fourier transforms of the raw and simulated time series were obtained using MATLAB’s “fft” function with a sampling frequency of 0.5 Hz, to determine the range, profile, and peak frequencies (Ritter et al., 2013). Phase: this was assessed by comparing the functional connectivity matrices of the simulated and empirical time series. We averaged all matrices from healthy controls to obtain a group control matrix, and calculated the pairwise linear correlation coefficient between the simulated functional connectivity matrix for each individual to the group.

(7) Differences in parameter values between healthy controls and stroke cases were evaluated with Wilcoxon sum rank test corrected for multiple comparisons (Bonferroni).

(8) Relationship with clinical phenotype. To determine whether there was any relationship between TVB parameters and the clinical phenotype, multiple linear regression was performed between model parameters (dependent variables) and the following independent variables: motor outcome measures (Fugl–Meyer, WMFT, 9-hole peg, and MAL-14), patient demographics (age, sex, presence of depression), and lesion characteristics (size, location, time after stroke, side of stroke).

## Results

### Weights of structural connections after stroke

**Figure 4.**
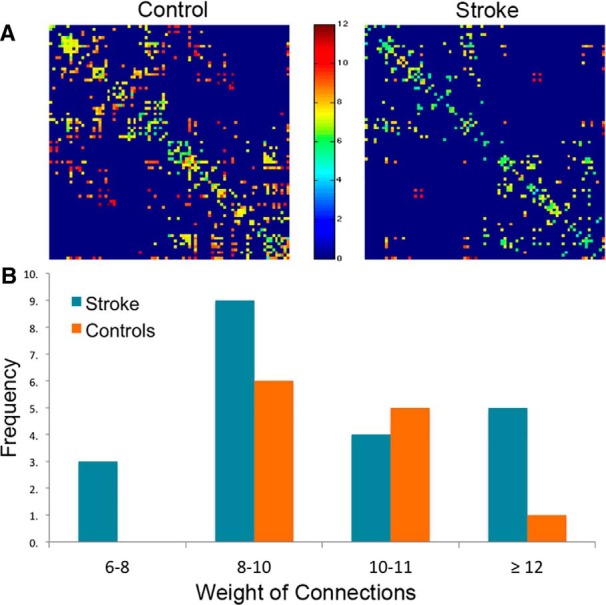
Weights of structural connections in stroke and healthy controls. ***A***, Structural connectivity matrices in a healthy control (left) and one individual with stroke (right). Dark blue denotes absence of connections while hotter colors indicate stronger weights. ***B***, Frequency distribution of weight of connections in healthy controls (orange bars) and stroke (blue bars).

The weights of connections in the control group had a mean (±SD) of 10.16 ± 1.03, (range, 8.75–12.07), and in the stroke cohort had a mean of 9.76 ± 1.57 (range, 6.41–10.35; Fig. 4). Yet, there were no statistical differences in mean, distribution shape between the groups (Kolmogorov–Smirnov test; *p*_a_ = 0.42), or skewness (controls = −0.083; stroke = −0.082; t test, *p*=0.35 and 0.29, respectively).

### BOLD simulations generated with TVB correlated with the empirical BOLD responses

**Figure 5.**
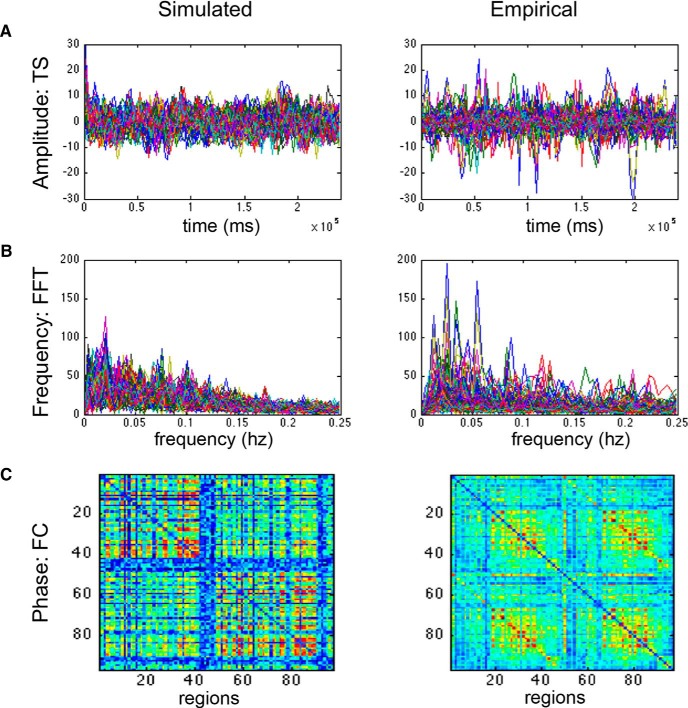
Comparison of simulated and empirical BOLD signals. ***A***, Amplitude: example of a raw simulated (left) and empirical (right) time series (TS). Amplitudes are indicated by the maxima and minima of the time series. ***B***, Frequency: frequency distribution graphs (FFT) of the simulated (left) and empirical (right) time series. Note that both empirical and simulated signals have the same range, profiles, and peaks. ***C***, Phase: functional connectivity (FC) matrix based on simulated time series (left) and the empirical group matrix (right).

The frequency spectrums of the simulated and the empirical BOLD responses had similar ranges (0–0.25 Hz) and mean peak (empirical = 0.05 + 0.035 Hz; simulated = 0.03 + 0.023 Hz; Fig. 5). Although the mean amplitudes were similar (empirical = 8.15; simulated = 9.49), the range of values was wider in the empirical signals (0.17–87.43) than those found in the simulated BOLD (3.79–22.64). The relative phases of the regions within simulated and empirical time series were similar as assessed by the mean correlation coefficient between their respective functional connectivity matrices (mean = 0.27±0.02; *p*_b_ = 0. 9e-12 Fisher *Z*-transformation). These validated simulations provided us with specific parameter values at both the global and the local levels associated with healthy control subjects and after stroke.

### Stroke was associated with reliable changes in global and local parameters

Although qualitative in nature, the color-coded graphic representation of the variance distribution done as part of the parameter space exploration (Table 3; Fig. 3) provides a glimpse into differences of combined values for the two global parameters: global coupling (*x*-axis) and conduction velocity (*y*-axis) in healthy controls and in stroke subjects, with warm colors representing higher variance. These explorations demonstrated at this early stage of analysis that the range of optimal parameter values (hot colors) in controls had similar topology of the distribution of variance, as well as concrete values. In contrast, stroke cases displayed high variations in both topology and values, where although some had similar distribution patterns as the healthy controls, others had scattered, fragmented patterns. Similar observations were found with respect to local parameters (Table 4).

**Table 3.** Summary of long-range and local parameters used in TVB to simulate BOLD time series in healthy controls and individuals with stroke

Group	Variable	Range	Mean	SD	Wilcoxon rank sum, *p*
**Control**	Global variables:				
	Global coupling	0.044–0.047	0.053	0.009	
	Conduction velocity	45–90	61.9	9.9	
	Model variables:				
	*K*_12_	0.12–0.55	0.49	0.338	
	*K*_21_	0.3–0.9	0.804	0.17	
	*K*_11_	0.6–0.95	0.833	0.142	
**Stroke**	Global variables:				
	Global coupling	0.04–0.09	0.061	0.016	0.013
	Conduction velocity	12–80	46	21	0.05
	Model variables:				
	*K*_12_	0.1–0.8	0.369	0.257	0.17
	*K*_21_	0.1–0.9	0.674	0.302	0.01
	*K*_11_	0.1–0.99	0.613	0.301	0.1

**Table 4.** Statistical table

	Comparison of interest	Data structure	Type of test	*p*
**a**	Weights of connections: stroke vs control	Normal	>Kolmogorov–Smirnov test	0.42
**b**	Pearson’s correlation coefficients: simulated vs empirical functional connectivity matrices	Normal after *Z*-transformation	*t* test	0.9e-12
**c**	TVB parameters: stroke vs control	Control: non-normal Stroke: normal	Wilcoxon rank sum test	Conduction Velocity: 0.05
				Global Coupling: 0.013
				*K*_12_: 0.17
				*K*_21_: 0.01
				*K*_11_: 0.1
**d**	Regression: TVB parameters with subject demographics, lesion characteristics and recovery	Normal	Multiple linear regression	Post-therapy: *K*_12_, Fugl–Meyer: 0.038
				Maintenance: *K*_12_, Fugl–Meyer: 0.005 Global coupling, WMFT: 0.039

*p*, Probability resulting from the Wilcoxon sum rank test comparing parameter values between the two groups.

Numerically, differences in parameter values between healthy controls and the stroke cohort are as follows:

#### Global parameters

(1) Conduction velocity: the range of modeled conduction velocities obtained via TVB in healthy controls ranged from 45 to 90 m/s with a mean of 62 ± 10 m/s. In contrast, the conduction velocities in stroke subjects had a range between 12 and 80 m/s with a mean of 46 ± 21 m/s. Comparison between the two groups with Wilcoxon rank sum test (*p*_c_ = 0.05) was marginally significant after correction for multiple comparisons.(2) Global coupling (rescale factor of incoming activity linking global with local dynamics): in healthy controls, the mean was 0.053 ± 0.009 (range, 0.044–0.047) and in cases with stroke the mean was 0.061 ± 0.016 (range, 0.04–0.09). Wilcoxon sum rank test showed this difference was significant after correction for multiple comparisons (*p*_c_ = 0.013).

In addition, it is important to note that the trend in all stroke cases where the values were different from those in controls was consistent: that is, it presented always as a decrease in conduction velocities (*N* = 12) and an increase in global coupling (*N* = 14). The rest of the stroke cases did not show differences with healthy controls.

#### Local parameters derived from the Stefanescu-Jirsa3D model

(1) *K*_12_ (coupling of excitatory over inhibitory populations within brain regions): the values of *K*_12_ in controls had a mean of 0.49 ± 0.338 (range, 0.12–0.55) and in stroke the mean was 0.369 ± 0.257 (range, 0.1–0.8). Statistical comparison between the two groups resulted in *p*_c_ = 0.17.

(2) *K*_21_ (coupling of inhibitory over excitatory populations): this variable (control mean = 0.804 ± 0.17; range, 0.3–0.9) was significantly reduced in the stroke group (mean = 0.674 ± 0.302; range, 0.1–0.9; *p*_c_ = 0.01).

(3) *K*_11_ (influence between excitatory populations): the values of *K*_11_ in controls had a mean of 0.833 ± 0.142 (range, 0.6–0.95) and in stroke cases had a mean of 0.613 ± 0.301 (range, 0.1–0.99). Comparison between the two groups with Wilcoxon sum rank test gave *p*_c_ = 0.1.

In summary, compared to values in healthy controls, there was a higher global coupling and a decrease of local inhibitory dynamics represented by the local parameter *K*_21_ along with a trend toward a reduction of conduction velocity.

### Global and local parameters were correlated with clinical phenotype

Multiple linear regression analysis to establish a relationship between modeling parameters and some clinical metrics did not show a correlation. The following clinical elements were considered in this preliminary assessment: stroke phenotype (size, location, time after stroke, side of stroke), depression, patient demographics (age, sex), and severity of impairment.

**Figure 6.**
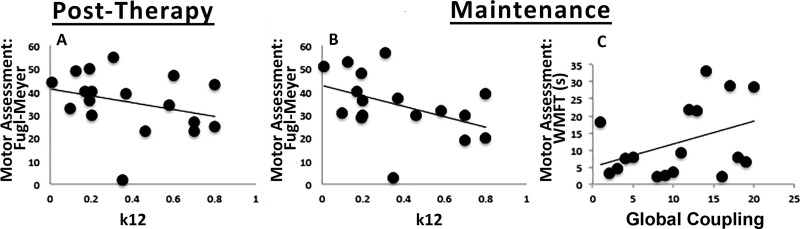
Correlation between modeling parameters and post-therapy motor outcomes. Scatterplots showing correlation between TVB modeling parameters (*x*-axis) and post-therapy motor outcomes (*y*-axis). Clear relationships were found between (***A***) *K*_12_ and Fugl–Meyer (Post-Therapy), (***B***) *K*_12_ and Fugl–Meyer (Maintenance), and (**C**) Global coupling and WMFT (Maintenance).

Next, we assessed the relationship between parameter values with recovery from stroke immediately after therapy and after 1 year (maintenance) using a multiple linear regression. This analysis showed a negative relationship between *K*_12_ and Fugl–Meyer scores both post-therapy (*t* =×2.386; *p*_d_ =0.038) and at maintenance 1 year later (*t* =−3.824; *p*_d_ =0.005). In addition, global coupling had a positive relationship with the Wolf Motor Function Test (*t*= 2.461; *p*_d_ =0.039) at maintenance. Thus, these two parameters derived from modeling based on pre-therapy conditions were related to long-term motor gains rather than the physical features of the stroke or the patient’s demographics (Fig. 6).

## Discussion

The main result of the study showed that the simulation of BOLD signals using TVB in stroke enables the identification of key changes associated with large-scale neural dynamics in individual patients. Overall, our results showed that, compared to healthy controls, individuals with stroke have a consistent reduction in conduction velocity and a relative increase in local-over-global brain dynamics. Further, the identified parameters were related to functional outcomes such that these parameters predicted long-term recovery after therapy. Together, these results not only back TVB as an effective tool in identifying dynamic brain changes in stroke spanning multiple scales, but also specifically identify potential predictors of recovery in stroke at the individual level. This study suggests that TVB may be a powerful platform for the application of large-scale modeling in understanding brain mechanisms at an individual subject level.

### Stroke is related to consistent global and local parameter changes

The successful simulation of empirical rfMRI data in this study facilitated a particularly salient finding; the dynamic model derived from stroke subjects had a significant decrease in the local parameter *K*_21_ and a consistent global coupling increase, accompanied by a trend in decreased conduction velocity. Two aspects of these results are of special interest: the first relates to the nature of the statistical outcomes and the second to the biological interpretation of these changes.

(1) Imaging-derived metrics in humans in general have high variance (Mueller et al., 2013); consequently, analytical measures have been developed to minimize it (Fischl et al., 1999). Further, this variance is amplified by stroke (Rehme et al., 2012), and has compelled researchers to stratify patients with precise criteria (Cramer, 2010), resulting in low sample sizes and high inter-study variability. In contrast, even when we used minimal exclusion criteria when selecting participants, changes seen after stroke were highly consistent, where all the cases that had a parameter change with respect to controls had the same directionality and relatively low variance. Given the high level of subject variability (as expected for a cohort including a large range of clinical phenotypes), we find this consistency somewhat surprising. However, we are not suggesting high reliability of our modeling, as the definitive answer will result from expanding the assessment to a larger population where the predictive value of the parameter changes can be formally assessed.

(2) Stroke survivors exhibited a significant decrease in *K*_21_, a parameter at the mesoscopic level that represents the influence of inhibitory on excitatory neuronal populations. A decrease in *K*_21_ thus indicates local disinhibition. These results are highly consistent with existing data on the basic mechanisms of stroke at the cellular level. For example, rodent models of MCA stroke show an imbalance in the density of excitatory and inhibitory receptors in tissue surrounding the lesion (Schiene et al., 1996). Specifically, they suggest a decrease in gamma-aminobutyric acid receptor expression in widespread ipsilesional cortical areas and a concomitant increase of N-methyl-D-aspartate receptor expression in the contralesional hemisphere.

In the context of stroke in humans, hyperexcitability has been described in two experimental paradigms:

(1) Studies using TMS to test cortical excitability after stroke have shown a decrease in the current needed to elicit motor evoked potentials and an increase in their amplitude (Hallett, 2007) along with an expansion in the area producing them (Liepert et al., 2000) suggesting disinhibition in motor cortices (Shimizu, 2002). Furthermore, decreasing the hyperexcitability via repetitive low-frequency stimulation (Takeuchi et al., 2005) along with a reduction of the TMS stimulation area (Liepert et al., 2000) has been related to motor recovery (Hallett, 2007).

(2) Increased activity in motor and non-motor regions has been reported in fMRI studies after stroke (Rehme and Grefkes, 2013). Specifically, increased contralesional activity has been observed (Weiller et al., 1992; Ward, 2003; Grefkes et al., 2008). Although this has been explained as a recruitment of supplementary areas to assist movement (Rehme and Grefkes, 2013), others have related it to widespread cortical hyperexcitability (Buchkremer-Ratzmann et al., 1996), suggesting long-range corticocortical inputs (Logothetis et al., 2001) with increased activation via decreased inhibition (Liepert, 2003; Blicher et al., 2009). Functional recovery has in turn been associated with the degree of recovery of activity in the affected cortical areas (Cramer, 2008).

Complementing the above, our results show a correspondence between local and global levels. Indeed, the reduction in local inhibitory influence over excitatory populations was accompanied by an increase in global coupling, reflecting an imbalance after stroke between global and local brain dynamics, favoring the latter. That is, local dynamics exert a stronger influence than global dynamics following stroke. In this case, the imbalance could be exacerbated by the decrease in conduction velocity. Interestingly, this imbalance has also recently been modeled in other brain diseases. For example, early stages of schizophrenia have been associated with a breakdown of local dynamics occurring prior to the disruption of global dynamics occurring later on in disease progression (Rubinov et al., 2009; van den Berg et al., 2012).

A particularly interesting finding was the trend associated with a decrease in conduction velocity in individuals with stroke, as it has previously been described through measurements of central motor conduction times (CMCTs) via transcranial magnetic stimulation (TMS) in the primary motor cortex. Immediately following stroke, CMCT decreases and correlates with functional measures (Abbruzzese et al., 1991; Pennisi, 2002) tending toward an incomplete normalization over the long-term (Heald et al., 1993). That being said, there is a paucity of information on decreased conduction velocity on corticocortical connections. The bulk of knowledge derives from studies in rodents showing structural changes to axons and oligodendrocytes in the primary lesion and the ischemic penumbra (Rosenzweig and Carmichael, 2015). In addition, although some degree of remyelination occurs in the recovery phase, the process is often arrested before completion (Syed et al., 2008). In human autopsy samples, there is an increase in nodal and paranodal lengths adjacent to lacunar lesions (Hinman et al., 2015), which may lead to decreased conduction velocities (Rasband, 2011). Our results thus provide direction for future animal studies, exemplifying the translational nature of TVB findings.

TVB thus appears to be effective at modeling brain activity in healthy brains and those impacted by disease processes, and has the novel capability of studying brain dynamics at multiple scales, including at a level that has thus far only been available via animal models or surrogate neuroimaging markers in humans. Applying this method of modeling, which is tied directly to biological mechanisms, to existing large datasets opens up the possibility to experiment with expanded models of brain states, including a myriad of diseases and their potential treatments.

### Potential predictors of motor recovery after stroke

Our results demonstrated that local (*K*_12_) and global (global coupling) parameters, derived from pre-therapy conditions, were significantly correlated with motor gains post-therapy and at maintenance. Furthermore, both parameters point in the same direction, as poor recovery was associated with an increase in local excitatory influences and with an emphasis on local dynamics, whereas values closer to controls correlated with better recovery.

Interestingly, TVB parameters in stroke did not correlate with severity of disease at the pre-stroke time point, even though the structural connectivity matrix used in the modeling coincided with this time point. In addition, other physical features of the stroke (size, location) or patient demographics (sex, age) did not correlate with the modeled parameters. Finally, neither lesion characteristics nor patient demographics correlated with recovery, highlighting the unique predictive potential of these parameters.

The question then becomes to what extent these parameter estimates can be used as predictors of recovery at the individual patient level. Although a cross-validation approach using the current dataset could serve to answer this question, a new and larger stroke cohort is ideal in obtaining estimates of the sensitivity and the specificity of our markers, due to high variance in stroke. However, there is clear value of our observations even with this limitation. At present, biomarkers for stroke recovery have been limited by the use of “substitute or surrogate” measures derived from brain imaging or electrophysiology, mainly due to the inability to measure *in vivo* more ideal basic elements, ie, at molecular or cellular levels (Burke and Cramer, 2013). Indeed, such elements may be observed more closely in animal models, but are difficult to translate to humans due to the limited homology between species. Specifically, the Stefanescu-Jirsa 3D model used in this study evolved from the mesoscopic level Hindmarsh–Rose model. The Hindmarsh–Rose model itself is rooted in the principles of the Hodgkin–Huxley neuron equations, in addition to dynamics based on bursting neurons found in invertebrate circuitry (Hindmarsh and Rose, 1984). Further, the neural behaviors described by the Hindmarsh–Rose model have been biologically verified in other animal models (Selverston and Ayers, 2006; Gu, 2013). Therefore, although any model of the mesoscale does not encompass the complexity of brain processes at the cellular level, there is likely emergence of behavior from the cellular level to the mesoscopic level, exhibiting deterministic behavior that can be modeled and also observed *in vivo*.

That is, the transition between the macroscopic and microscopic level is represented by population dynamics at the mesoscopic level (Mitra, 2014). From this, one could conclude that the path toward basic biomarkers should include the intermediate mesoscopic level. Indeed, TVB allows one not only to estimate parameters at that level but also to link it to the macroscopic global whole-brain level. TVB is not unique in considering biophysical parameters as exemplified by inference models based on DCM (Moran et al., 2011). Basically, there are no conceptual differences in the inferential goals between TVB and DCM but they do differ in the detailed mechanics. For example, whereas TVB develops the model at the level of large-scale networks, DCM focuses on portions of these networks. Second, and perhaps the key contrast is that whereas DCM fits the parameter of the model but does not generate data, TVB uses the model to generate data, making these two approaches highly complementary.

An interesting and unique aspect of TVB is its highly individualized approach, as parameter estimates are derived from individualized structural connectivity matrices obtained from each subject, and hence, it can provide the first step to customize individual therapeutic interventions. For example, our ongoing work is beginning to test potential “virtual interventions” by modifying specific parameters changed after stroke and determining the degree of restoration of brain dynamics on each stroke patient.

A second ability of this modeling approach is to use the model of an individual patient’s brain connectivity that can be objectively measured and evaluated as an indicator of normal biological processes (such as, resting state activity, rsfMRI), pathogenic processes, or pharmacologic responses to therapeutic intervention (Biomarkers Definitions Working Group, 2001). Dynamics of rsfMRI are highly nonstationary (Allen et al., 2014) and existing metrics, including the direct correlation between functional and structural connectivity, are so far incapable of addressing this issue satisfactorily (Goni, 2014). A number of studies have therefore used generative modeling to parse the relationship between structural and functional connectivity. A recent study (Andersen et al., 2014) demonstrated that the fusion of TVB-like network modeling with structural neuroimaging explains the nonstationary dynamics observed in rsfMRI. Thus, we propose a conceptual paradigm shift, in which the dynamic model shifts the nonstationary functional data from imaging at the mesoscopic scale to a more deterministic set of coefficients in a brain model. In other words, complex dynamics cannot be captured by stationary imaging analyses, but can be generated by a data-constrained mechanistic model of brain- circuit dynamics, as seen in the generative modeling approach detailed in stroke (Brodersen et al., 2011). Thus, the mathematical model could be seen as a compact generator of dynamics-based biomarkers, or even as the biomarker itself. The primary benefit, as we demonstrated here, is that it becomes easier to understand disease mechanisms by evaluating the coefficients of the model.

Of note, the approach used in this study to validate the simulated time series was to compare frequency, amplitude, and phase of the simulated and empirical signals. After the refinement of the TVB models, future studies will incorporate a larger variety of multidimensional analyses, particularly with respect to temporal variability in resting state signals. Furthermore, the current study determined optimal values of local parameters applied to all brain regions. Future studies will focus on local parameters for subsets of brain regions, eg, changing parameters of nodes within and/or around a stroke lesion to determine how this impacts the resultant simulated brain activity. We also note that the translational power of our findings depends upon the reproducibility of parameters for a given brain state, the answer for which will emerge with expanded application of TVB to other cohorts. The results from this study thus confirm that TVB allows the assessment of biophysical variables previously unattainable in human studies. This method provides a potentially important and novel application of large-scale modeling, in which we can probe brain dynamics and biomarkers on an individual level. Therefore, The Virtual Brain has the potential to become an important step toward the development of individualized medicine in stroke.

## Synthesis

The decision was a result of the Reviewing Editor Gustavo Deco and the peer reviewers coming together and discussing their recommendations until a consensus was reached. A fact-based synthesis statement explaining their decision and outlining what is needed to prepare a revision is listed below. The following reviewers agreed to reveal their identity: Klaas Enno Stephan, Adrian Ponce-Alvarez

Both Reviewers are very positive and stress some points that I believe are reasonable and would improve the final version of the paper, so please try to answer/fulfill them. Both Reviewers are very positive and stress some points that I believe are reasonable and would improve the final version of the paper, so please try to answer/fulfill them.

Reviewer #1:

"Functional mechanisms of recovery after chronic stroke: modeling with The Virtual Brain"

This is a very interesting paper that addresses an important and timely topic. I am very sympathetic to the overall approach, but also have a few suggestions that you may wish to consider in a revision of your paper.

Major points

1. Several aspects of the procedure by which parameter values were determined were not entirely clear to me.
2. Page 10: "... significant similarity between the simulated signals and the empirical signals..." - what exactly was the statistical measure of similarity here, and how was significance defined?
3. Page 10: "Highest overall variance" - of what? Over multiple integration of the state equations? Similarly, in the legend of Figure 3, what is "global variance" referring to?
4. Related to the previous point, I did not understand how I should interpret Figure 3. Could you please explain this in more detail and give an example how you determine an optimal parameter value from these plots? To what degree are optimal parameter values unique, and how is this operationalised?

1. Following from the previous point, a brief section in the discussion on why the present parameter exploration procedure was chosen instead of fitting the models against empirically observed data (which might be possible, given the relatively low number of global parameters?) would be extremely helpful for many readers. Is it simply practical constraints, such as runtime, that lead you to favour exploration over fitting?
2. The statistical tests of differences in parameter values between controls and patients depend on the reliability (observer independence) of parameter choices. I think a cautionary note in this regard would be appropriate. I did note that you suggested that the pattern of results (same directionality of change in the subset of patients, which showed differences to controls) indicated high reliability. However, I must confess that I do not find this entirely convincing, unless quantified by a statistical analysis of how likely this pattern is to occur, in reference to an appropriately defined null hypothesis.
3. The differences in parameter values between patients and controls are very interesting, and I agree that many of them make sense. One major exception is the decrease in conduction velocity you found. Biologically, this could only happen if, following stroke, either average axon diameter would decrease or the degree of myelination would be reduced. I am not aware of evidence for either option (and the first scenario generally seems rather implausible to me). I would be curious to hear your thoughts on this?

Minor points

1. Page 4: "... computational microscope, allowing the inference of internal states..." - it might be helpful to qualify this statement in relation to the statistical concept of "inference" and generative models. For example, what are the similarities and differences of your model compared to the type of "mathematical microscope" described by Moran et al 2011 (Current Biology)?
2. Page 7: "... parcellated into 96 cortical and subcortical regions based on the MNI brain template..." - by itself, the MNI template does not suggest any parcellation, could you clarify?
3. You refer to "big data" several times through the manuscript. I wonder whether this wording is appropriate, and whether a slightly more sober term would not serve your work far better. I say this because the analysis you present does not seem to match a typical "big data" approach, which usually rests on searching for statistical relations in large data sets, and without guidance by mechanistic concepts. On the contrary, you do something I find much more convincing, i.e., you present a carefully developed model which condenses a high dimensional (but fairly standard sized) imaging dataset to a few interpretable parameter estimates.
4. "In order to get estimates of the sensitivity and the specificity of our markers, a new stroke cohort is needed." - not necessarily, you could also resort to a cross-validation approach on the present data?
5. Page 21: "... the first step to customise individual therapeutic interventions targeting specific physiological mechanisms..." - could you be more specific here? To what degree do the present parameters allow for choosing between available therapeutic approaches in stroke?
6. Page 22: "Thus, the mathematical model could be seen as a compact generator of dynamics-based biomarkers, or even as the biomarker itself." I thought this was a beautiful summary and would like to point out that this perspective connects your approach to the "generative embedding" approach, which was introduced by Brodersen et al. 2011 (PLoS Computational Biology) and demonstrated using stroke as an example.

I hope you will find these thoughts and suggestions helpful and look forward to your response.

Reviewer #2:

The present study is an important contribution that combines theoretical work together with structural, functional, and behavioral data from controls and stroke patients. The authors used The Virtual Brain, a platform that is a promising tool that combines empirical data and large-scale models of the brain to unravel brain mechanisms at an individual subject level. The work is timely and well written (although I think that the figures could be improved). The conclusions of the study are reasonable and justified. Nevertheless, I think the manuscript needs some few clarifications:

1. The agreement between the empirical functional connectivity (FC) and the model FC is equal to 0.27 on average (+/-sd should be added). This value is reasonable when using individual data. However, I would like to see the correlation between the structural connectivity and the FC, in order to know how much the dynamics add to the fitting.
2. I am not sure whether the global coupling (c) and the local inhibition (K21) are fully independent. Different nodes are connected through the variable x (field potential) which seems to be a combination of excitation and inhibition (this is not clear in manuscript). If this is the case, it's possible that c and K21 are correlated.
3. A final test would be to increase c and decrease K21 in the model estimated from the control data and see whether this affects the time series in the same way that a stroke.
4. the authors show that local excitation-to-inhibition (K12) negatively correlates with motor gains. This seems to me contradictory with the result that the inhibition K21 is decreased in stroke patients. An increase of K12 could compensate the lack of inhibition and improve performance, but a decrease would be detrimental. So a positive correlation would be expected. When correlating the parameters and the task performance the values of the correlation coefficients should be shown, not only the p-values.
5. At last, the local parameters K are the same for all nodes. But, in first approximation, strokes are localized, so how can the local parameters of all brain regions change?

Other comments:

- The authors used short simulations (4 min, at 2s sampling) to have the same points as in the data. In my opinion, they should use longer simulations to have better estimates. The model doesn't need to have the same limitations as the empirical data. What is the justification for this choice?

-line 174: "covariance" -&lt; "correlation".

-line 294: " +/-0 ", the uncertainty can't be 0, what is the step used for the parameter variation?
